# The Origins of Specialization: Insights from Bacteria Held 25 Years in Captivity

**DOI:** 10.1371/journal.pbio.1001790

**Published:** 2014-02-18

**Authors:** Vaughn S. Cooper

**Affiliations:** Department of Molecular, Cellular, and Biomedical Sciences, University of New Hampshire, Durham, New Hampshire, United States of America

## Abstract

Examples of ecological specialization abound in nature but the evolutionary and genetic causes of tradeoffs across environments are typically unknown. Natural selection itself may favor traits that improve fitness in one environment but reduce fitness elsewhere. Furthermore, an absence of selection on unused traits renders them susceptible to mutational erosion by genetic drift. Experimental evolution of microbial populations allows these potentially concurrent dynamics to be evaluated directly, rather than by historical inference. The 50,000 generation (and counting) Lenski Long-Term Evolution Experiment (LTEE), in which replicate *E. coli* populations have been passaged in a simple environment with only glucose for carbon and energy, has inspired multiple studies of their potential specialization. Earlier in this experiment, most changes were the side effects of selection, both broadening growth potential in some conditions and narrowing it in others, particularly in assays of diet breadth and thermotolerance. The fact that replicate populations experienced similar losses suggested they were becoming specialists because of tradeoffs imposed by selection. However a new study in this issue of *PLOS Biology* by Nicholas Leiby and Christopher Marx revisits these lines with powerful new growth assays and finds a surprising number of functional gains as well as losses, the latter of which were enriched in populations that had evolved higher mutation rates. Thus, these populations are steadily becoming glucose specialists by the relentless pressure of mutation accumulation, which has taken 25 years to detect. More surprising, the unpredictability of functional changes suggests that we still have much to learn about how the best-studied bacterium adapts to grow on the best-studied sugar.

The wonder of biological diversity belies a puzzling subtext. Species are defined as much by their limits as their capabilities. Very few species in our common vernacular tolerate life in a wide range of environments, and those that do—the Norway rat, say—are not generally appealing. More often, we celebrate specialization to a particular condition: for example, orchid epiphytes growing tenuously in the cloud forest, only a subtle climate shift from extinction. Even grade school natural history teaches us that species are often unfit when living beyond their natural range.

So it comes as a surprise that the causes of this rampant ecological specialization are poorly understood. “Use it or lose it,” but why? One common explanation is that natural selection tends to favor traits that simultaneously enhance fitness in one environment but compromise fitness elsewhere. This selective process is known as “antagonistic pleiotropy.” Another explanation is that a selective shadow falls upon unused traits, rendering them susceptible to mutational erosion by random genetic drift. This neutral process is known as “mutation accumulation” (Figure 1). These processes inevitably co-occur, and can be enhanced by the hybrid dynamic of genetic hitchhiking, in which neutral mutations affecting unused functions become linked to different mutations under positive selection. In most cases, the functional decay of a species can only be studied retrospectively, and distinguishing the roles of antagonistic pleiotropy and mutation accumulation is hampered by weak historical inference. Did selection, or an absence of selection, produce the blind cavefish [1]? There is little controversy that the sum of these dynamics can produce specialists, but their timing and relative importance is an open question.

**Figure 1:**
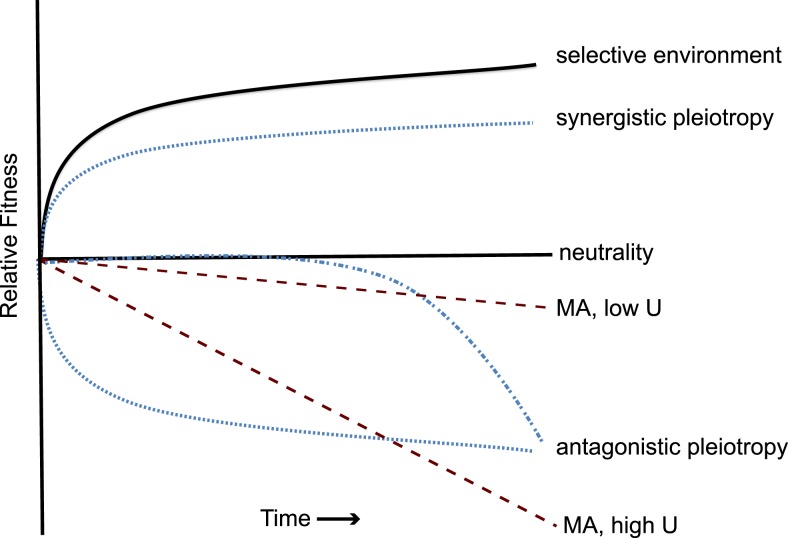
Hypothetical dynamics of fitness in foreign environments by pleiotropy or mutation accumulation during long-term adaptation. Prolonged adaptation to one environment leads to decelerating fitness gains in the selective environment (solid black line), as beneficial mutations become limiting. Consequences of this adaptation for fitness in other environments may take different forms. No net change may occur if beneficial mutations generate no or inconsistent side effects (neutrality). However, the same mutations responsible for adaptation may also increase fitness in other environments (synergistic pleiotropy, dotted line), may decrease fitness in foreign environments at an equivalent rate if antagonistic effects correlate with selected effects (antagonistic pleiotropy, dotted line), or may decrease fitness at an increasing rate if subsequent mutations generate greater tradeoffs (antagonistic pleiotropy, dashed and dotted line). The uncertainty of the form of pleiotropic effects reflects a general lack of understanding of how mutations interact to affect fitness, particularly over the long term. Mutation accumulation (MA) in traits hidden from selection is expected to reduce fitness randomly but linearly on average, more slowly during evolution at a low mutation rate (MA, low U) or more rapidly at a high mutation rate (MA, high U). Evidence of all processes is now evident in this latest study of the evolution of diet breadth in the LTEE [20].

The study of “evolution in action” using model experimental populations of rapidly reproducing organisms allows researchers to quantify both adaptation and any functional declines simultaneously. This approach is especially powerful when samples of evolving populations can be stored inanimate and studied at a later time under various conditions. Perhaps the best example of this approach is Richard Lenski's Long-Term Evolution Experiment (LTEE), in which 12 populations of *E. coli* have been grown under simple conditions for more than 25 years and 50,000 generations [2],[3].

When as a graduate student I wondered aloud whether the LTEE lines had become specialists, a colleague remarked: “Of course! You've selected for streamlined *E. coli* that have scuttled unused functions.” But with only a small amount of glucose as the sole carbon source available to the ancestor (the innovation by one population of using citrate for growth more than 30,000 generations in the future notwithstanding [4]), all anabolic pathways to construct new cells remain under strong selection to preserve their function. Moreover, because some catabolic reactions use the same intermediates as anabolic pathways (a form of pleiotropy) [5], growth on alternative carbon sources may be nonetheless preserved. Thus, we wondered whether the physiology of *E. coli* might actually prove to be robust during long-term evolution on glucose alone.

Over the first 2,000 generations, the LTEE lines gained more often than lost fitness across a range of different environments [6]. In addition, a high-throughput screen of cellular respiration (Biolog) for the best-studied clone from these lines showed 171 relative gains and only 32 losses [7]. Even these losses in substrate respiration did not translate to reduced fitness versus the ancestor; rather, the evolved clone was simply relatively worse in the foreign resources than in glucose [7]. Evidently, each of the five beneficial mutations found in this early clone was broadly beneficial and imparted few tradeoffs [8]. Generalists rather than specialists were the rule.

Between 2,000 and 20,000 generations, fitness losses in foreign conditions became more obvious but not always consistent. Some lines became less fit than the ancestor in a dilute complex medium (LB) [9], all lines grew worse at high (>40°C) and low (<20°C) temperature [10], and all lines became sensitive to the resource concentration in their environment, even for glucose [9]. Did subsequent beneficial mutations cause these tradeoffs (antagonistic pleiotropy), or did other, neutral or slightly harmful mutations accumulate by drift (Figure 1)? We must consider the population genetic dynamics of these LTEE populations. The hallmark of neutral theory [11] is that mutations with no effect in the selective environment should become fixed in the population at the rate of mutation. For the ancestor of this experiment, the mutation rate is ∼10^−3^ per genome per generation [12],[13], so only a handful of neutral mutations would have fixed by the time tradeoffs became evident, and would not likely explain the early specialization.

However, an important extension of neutral theory is that slightly harmful mutations—those whose effects are roughly the inverse of the population size or below, 1/N—can also be fixed by drift [14]. Millions of slightly deleterious mutations were produced in these populations, which cycled between 5×10^6^ and 5×10^8^ cells each day. Might these mutations account for tradeoffs over the first 10–20,000 generations? In small populations, the effect of these mutations can be substantial, which explains why bottlenecked populations may experience fitness declines or even the genome erosion frequently seen in bacterial endosymbionts [15]. But in the large LTEE populations, most deleterious mutations are weeded out by selection and only those with the slightest effects may accumulate over very long time scales. Thus, because these early losses tended to occur when adaptation in the selective environment was most rapid, and because the randomness and rarity of mutation accumulation should not produce parallel changes over these time scales, early specialization is best explained by antagonistic pleiotropy [9],[10].

Later in the LTEE, elevated mutation rates began to evolve in certain lines, resulting in a fundamental change in the population genetic environment [16],[17] that should increase the rate of functional decay in unused, essentially neutral functions. These mutator populations tended to perform worse in multiple environments, and in theory should continue to specialize more rapidly by accelerated mutation accumulation. As a first test, we used Biolog plates to assay respiration on 95 different carbon sources over the first 20,000 generations [18]. Although mutators tended to exhibit a reduced breadth of function in this assay, the difference was not statistically significant [18]. Rather, a surprising number of losses of function were shared among replicate lines, and we took this parallelism as further support of antagonistic pleiotropy driven by selection for common sets of adaptive mutations.

Here the LTEE offers its greatest advantage: more time, both for evolution and innovative research. Over subsequent generations, mutator lines should continue to accumulate greater mutational load by drift and hence become more specialized than lines retaining the low ancestral rate. Genomic sequences of the evolved lines now have confirmed this increased mutational load [3],[19] in the six of 12 lines that are now mutators [16]. In this issue, Leiby and Marx [20] have readdressed these questions by retracing old steps, applying the prior Biolog assays to lines spanning 50,000 generations of evolution, and by pioneering new high-throughput assays of fitness in many resources. Somewhat surprisingly, these methods disagree and challenge the reliability of Biolog data as a fitness proxy. As a proprietary measure of cellular respiration, it can demonstrate major functional shifts but is less reliable than growth rate as a fitness parameter.

More importantly, Leiby and Marx provide clear evidence that niche breadth in the LTEE was shaped by both mutation accumulation and pleiotropy. Growth rates actually increased on several resources, and hence the pleiotropic effects of adaptation to glucose were synergistic, broadening functionality particularly over the first 20,000 generations, as well as antagonistic, producing fewer tradeoffs than previously thought [20]. Pleiotropic effects were also somewhat unpredictable: a sophisticated flux-balance analysis [21] of foreign substrates did not reveal more gains for resources similar to glucose or losses for dissimilar resources. Some early losses linked to selection (maltose, galactose, serine) [6] became complete, but also subtle gains of function for dicarboxylic acid metabolism, perhaps related to growth on metabolic byproducts, became amplified. The most striking pattern was that mutator populations became specialists, diminished for many functions owing to their greater mutational burden, and this only became evident after 50,000 generations in a single resource. These convergent functional losses were not caused by selection, as is often argued, but rather by an absence of selection in the face of mutational pressure. Mutational decay by genetic drift takes a long time, and it will take much longer for the non-mutator lines, it seems.

Although Leiby and Marx [20] correctly emphasize the importance of truly long-term selection combined with deficient DNA repair to reveal effects of mutation accumulation, decay has been witnessed in other systems undergoing regular population bottlenecks over shorter time scales [22],[23]. Antagonistic pleiotropy can also reveal its effects much more rapidly than was seen in the LTEE, especially when selection discriminates among discrete fitness features in a heterogeneous environment, such as in the colonization of a new landscape [24],[25]. What this study uniquely illustrates is the unpredictability of pleiotropic effects of adaptation to a simple environment, which in turn shows how chance draws from a distribution of contending beneficial mutations may produce divergent outcomes, ranging from generalists to specialists. A sample of the first mutants competing to prevail in the LTEE system showed variable niche breadth [26] so perhaps we should not be surprised that the footprints of these large-effect mutations endure. Further study of the precise mechanisms by which different mutations produce more fit offspring will teach us more about the origins of diversity that beguile us. We can also gain a broader perspective on the longstanding tension between chance and necessity [27]—a motivator of the LTEE—by focusing more on what is unnecessary, such as how organisms grow in foreign environments. Often insight comes from studying at the margins of a problem, and here, the limits to the growth of these bacteria have allowed us to focus more on how exactly they have accomplished their most essential tasks.
